# A case report of PR-3-ANCA-positive glomerulonephritis with histological features of GPA associated with infectious endocarditis

**DOI:** 10.1097/MD.0000000000026905

**Published:** 2021-08-13

**Authors:** Momoko Hirata, Haruhisa Miyazawa, Junki Morino, Shohei Kaneko, Saori Minato, Yanai Katsunori, Hiroki Ishii, Taisuke Kitano, Kiyonori Ito, Keiji Hirai, Takashi Oda, Akira Shimizu, Yoshihiko Ueda, Yoshiyuki Morishita

**Affiliations:** a Division of Nephrology, First Department of Integrated Medicine, Saitama Medical Center, Jichi Medical University, Saitama, Japan; b Department of Nephrology and Blood Purification, Tokyo Medical University Hachioji Medical Center, Tokyo, Japan; c Department of Analytic Human Pathology, Nippon Medical School, Tokyo, Japan; d Department of Diagnostic Pathology, Dokkyo University Koshigaya Medical Center, Saitama, Japan.

**Keywords:** granulomatosis with polyangiitis, infectious endocarditis, proteinase 3-anti-neutrophil cytoplasmic antibody positive glomerulonephritis

## Abstract

**Rationale:**

Several renal diseases are associated with infectious endocarditis. However, there are few reports on patients with granulomatosis with polyangiitis (GPA) associated with infectious endocarditis, and there is no consensus for appropriate treatment.

**Patients concerns:**

A 35 -years-old man with congenital ventricular septal defect presented severe anemia, hematuria and proteinuria. The blood and urine examinations showed elevated white blood cells (12,900 cells/μL), C-reactive protein level (13.1 mg/dL) and proteinase 3-anti-neutrophil cytoplasmic antibody (PR3-ANCA) level (11.0 IU/mL), severe anemia (hemoglobin: 6.1 g/dL) and renal dysfunction [estimated glomerular filtration rate (eGFR): 12.7 ml/min.1.78 m^2^ with hematuria and proteinuria].

**Diagnoses:**

The patient was diagnosed with crescentic glomerulonephritis with histological features of GPA associated with infectious endocarditis by renal biopsy and transthoracic echocardiography.

**Interventions:**

Antibacterial drugs (ampicillin-sulbactam) were administrated. No immunomodulating agents were used because immunosuppressive drugs may worsen infectious endocarditis. Subsequently, renal function and urinary findings improved. However, infectious endocarditis was not improved. Therefore, valve replacements and ventricular septal closure surgery were conducted.

**Outcomes:**

Thereafter, his postoperative course was uneventful, renal function improved (eGFR: 64.3 ml/min.1.78 m^2^), and PR3-ANCA level normalized.

**Lessons:**

We reported a case report of PR3-ANCA positive glomerulonephritis with histological features of GPA associated with infectious endocarditis. Physicians might note this renal complication when they manage infectious endocarditis.

## 1 Introduction

Several forms of renal disease are accompanied with infectious endocarditis.^[1–3]^ Patients with infectious endocarditis can also develop multiple serological abnormalities including antineutrophil cytoplasmic antibody (ANCA) production, notably proteinase-3-ANCA (PR3-ANCA). Additionally, granulomatosis with polyangiitis (GPA) (previously known as Wegener's granulomatosis) is a small vessel vasculitis associated with ANCAs, especially PR3-ANCA. However, few reports have described patients with GPA associated with infectious endocarditis.

Herein, we describe a 35-year-old man who presented rapidly progressive crescentic glomerulonephritis with histological features of GPA associated with infectious endocarditis. The patient's renal biopsy revealed histopathological features of GPA. He was treated successfully with antibacterial monotherapy, and the titer of PR3-ANCA normalized with improvement of renal disease.

## 2 Case presentation

A 35-year-old man presented with congenital ventricular septal defect. Periodic medical examinations showed normal heart function that required no intervention. Additionally, to this point, he had no history of renal dysfunction such as proteinuria or hematuria. Although he was asymptomatic, renal dysfunction and severe anemia were identified upon routine medical checkup, and he was advised to be admitted to hospital. On admission, he had normal temperature at 36.3°C, he had elevated white blood cells [12,900 cells/μL (normal range: 3900–9800 cells/μL)] and C-reactive protein level [13.1 mg/dL) (normal range:0.00–0.14 mg/dL)], severe anemia [hemoglobin: 6.1 g/dL (normal range:12.0–17.6 g/dL)], and renal dysfunction [estimated glomerular filtration rate (eGFR): 12.7 ml/min.1.78 m^2^ with hematuria and proteinuria]. The blood culture was not conducted. He was treated with an antibacterial agent for suspected infection and transfusions for the anemia. However, his condition and laboratory parameters, including renal dysfunction, did not improve. Therefore, 3 days after admission, he was referred to our department for further management.

On admission, his body temperature was 36.7°C, and his blood pressure was 150/106 mm Hg. A systolic murmur was auscultated in the intercostal space at the left sternal border, and bilateral edema of the lower extremities was observed. There were no other physical findings of infectious endocarditis such as Osler's nodes, Roth spots, or Janeway lesions. Blood testing indicated renal dysfunction [stimated glomerular filtration rate (eGFR): 13.8 ml/min.1.78 m^2^, blood urea nitrogen: 54 mg/dL], inflammation [white blood cells: 14,960 cells/μL, C-reactive protein: 11.09 mg/dL, procalcitonin: 1.16 ng/mL (normal range: <0.5 ng/mL)], and anemia (hemoglobin: 8.8 g/dL). Urinalysis showed microscopic hematuria, and proteinuria (1.49 g/gCr). PR3-ANCA level was elevated at 11.0 IU/mL (normal range: <2.0 IU/mL), and antistreptolysin O was elevated at 367 IU/mL (normal range: 0–240 IU/mL). Hepatitis B antigen, hepatitis C antibody, antinuclear antibody, myeloperoxidase-ANCA, and antiglomerular basement membrane antibody were negative. Serum complement C3 [39 mg/dL (normal range: 86–160 mg/dL)] and CH50 [< 10 U/mL (normal range: 30–46 U/mL)] levels were decreased, while C4 [26 mg/dL (normal range: 17–45 mg/dL)] level was normal. Two blood cultures were negative. Detailed laboratory data on admission are shown in Table 1. Transthoracic echocardiography showed a vegetation on the pulmonary valve, and computed tomography showed multiple nodular shadows in bilateral lung fields, and bilateral kidney enlargement. The patient met the modified Duke criteria for definitive infectious endocarditis.

**Table 1:** Laboratory findings on admission.

Complete blood count and blood chemistry				Immunological testing			
		Unit	Normal range			Unit	Normal range
WBC	14960	/μL	3900–9800	ASO	367	IU/mL	0–240
Band	1.0	%	0–19	IgG	3239	mg/dL	870–1700
Segment	90.0	%	25–72	IgA	479	mg/dL	110–410
Eosinophil	0	%	0–7.0	IgM	315	mg/dL	33–190
Basophil	0	%	0–2.0	C3	39	mg/dL	86–160
Lymphocyte	4.0	%	19.0–49.0	C4	26	mg/dL	17–45
Monocyte	5.0	%	3.4–9.0	CH50	<10	U/mL	30–46
RBC	323	×10^4^/μL	427–570	ANA	40		(-)
Hemoglobin	8.8	g/dL	12.0–17.6	PR3-ANCA	11.0	IU/mL	<2.0
Hematocrit	27.9	%	39.8.–51.8	MPO-ANCA	<1.0	IU/mL	<3.5
Platelet	16.6	×10^4^/μL	13–36.9	Anti-GBM-Ab	<1.0	IU/mL	<7.0
Total protein	7.7	g/dL	6.6–8.1	**Urine analysis**			
Albumin	1.9	g/dL	4.1–5.1				
AST	18	IU/L	13–30			unit	Normal range
ALT	17	IU/L	10–42	Gravity	1.015		1.005–1.025
CRP	11.09	mg/dL	0.00–0.14	pH	5.0		
Na	131	mmol/L	138-145	RBC	30–49 (dysmorphic)	/HPF	<5
K	3.7	mmol/L	3.6–4.8	WBC	20–29	/HPF	<5
Cl	96	mmol/L	100–110	Protein	1.49	g/gCr	0.15
Ca	7.7	ng/dL	8.4–10.1	NAG	47.5	IU/L	<7
P	6.9	mg/dL	2.7–4.6	β2-MG	273	μg/L	<230
BUN	54	mg/dL	8–20	Granular casts	30–40	/WF	(-)
Cr	4.41	mg/dL	0.65–1.07	Waxy casts	1–4	/WF	(-)
eGFR	13.8	ml/min/					
		1.73m^2^					
Uric Acid	10.4	mg/dL	3.7–7.8				
HbA1c	5.3	%	4.6–5.2				
Glucose	94	mg/dL					
Ferritin	968.5	ng/mL	20-250				
TSAT	19.2	%	>20				
HBS-Ag	(-)		(-)				
HCV-Ab	(-)		(-)				
PCT	1.16	ng/mL	<0.5				

**Figure 1:**
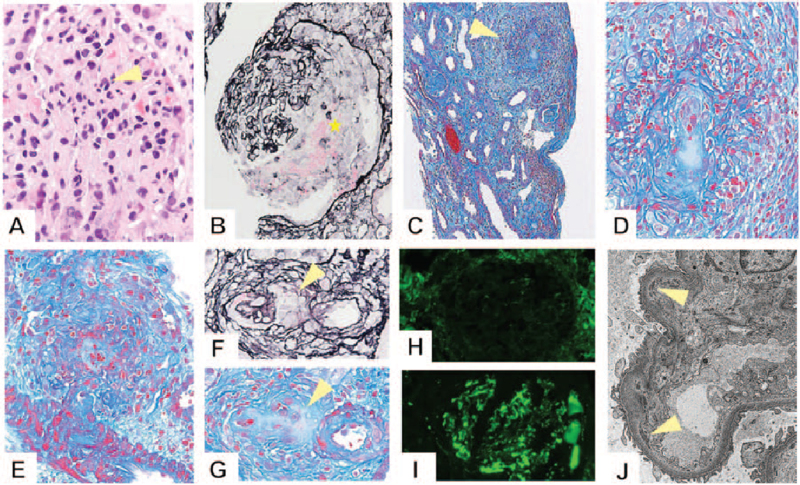
Renal biopsy findings. (A) Neutrophil infiltration within the glomerulus (hematoxylin-eosin stain; magnification, 600 × ). (B) Formation of cellular crescent (Periodic acid-methenamine-silver stain; magnification, 400 × ). (C) Necrotizing granuloma formation in the cortex area (Masson trichrome stain; magnification, 200 × ). (D) High magnified image of Fig. 1C (Masson trichrome stain; magnification, 400 × ). (E) Necrotizing granulomatous arteritis in arteriole (Masson trichrome stain; magnification, 600 × ). (F) Necrotizing and granulomatous arteritis in arteriole (Periodic acid-methenamine-silver stain; magnification, 800 × ). (G) Same area as in Fig. 1F (Masson trichrome stain; magnification, 800 × ). (H) No deposition of immunoglobulin G (immunofluorescence; magnification, 400 × ). (I) Granular complement component 3 staining on the mesangial areas and glomerular capillary walls (immunofluorescence; magnification, 400 × ). (J) Small electron-dense deposits in the subendothelial area (uranyl acetate lead citrate stain; magnification, 8000 × ).

**Figure 2:**
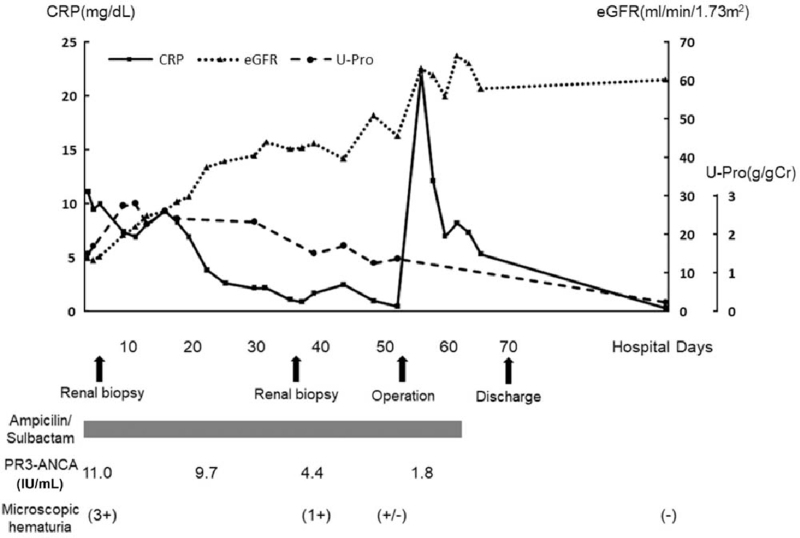
The patient's clinical course. CRP = C-reactive protein, eGFR = estimated glomerular filtration rate, PR3-ANCA = proteinase-3 antineutrophil cytoplasmic antibody, U-Pro = urinary protein.

On the second hospital day, we performed renal biopsy to evaluate his renal disease. Histological analysis revealed that 57% (8/14) of glomeruli showed cellular crescents (Fig. 1B, star) with intraglomerular neutrophil infiltration (Fig. 1A, arrowhead). Necrotizing granuloma formation was observed in the tubulointerstitial area (Fig. 1C, arrowhead). Figure 1D shows a high magnified image of Figure 1C. Granuloma formation around an artery was observed. Necrotizing granulomatous arteritis of arterioles was also observed (Fig. 1E). Arterioles demonstrating necrotizing vasculitis with fibrin exudation (Fig. 1F and 1G, arrowheads) and those with granulomatous arteritis without fibrin exudation were observed (Fig. 1F and 1G). Immunofluorescence microscopy showed granular C3 deposition along capillary walls in glomeruli without deposition of immunoglobulins or other complement factors (Fig. 1H and 1I). Nephritis-associated plasmin receptor (NAPlr) staining and plasmin activity on glomeruli were negative (data not shown). Electron microscopy showed small electron-dense deposits in subendothelial and paramesangial areas (Fig. 1J). On the basis of these findings, the patient was diagnosed with crescentic glomerulonephritis with histological features of GPA associated with infectious endocarditis. Although he did not have upper respiratory lesions, he met the diagnostic criteria for GPA (abnormal urinalysis, abnormal findings on chest imaging, granuloma on biopsy, and PR3-ANCA positivity). We began therapy with antibacterial drugs (ampicillin-sulbactam) only. No immunomodulating agents were used because immunosuppressive drugs may worsen infectious endocarditis. Subsequently, the inflammatory response decreased steadily, and renal function, the urinary findings and the levels of PR3-ANCA improved [CRP: 2.09 mg/dL, eGFR: 40.4 ml/min.1.78 m^2^, proteinuria 2.32 g/gCr, PR3-ANCA: 9.7 IU/mL] on hospital day 30 with antibiotic therapy for management of the endocarditis (Fig. 2). He did not require dialysis.

However, the vegetation on the pulmonary valve grew gradually, and an aortic regurgitation appeared. Therefore, valve replacements and ventricular septal closure surgery were required.

We repeated the renal biopsy on hospital day 36 to re-evaluate his renal pathological condition before receiving heart operation. According to histological analysis, 31% (4/13) of glomeruli showed fibrous or cellular crescents, GPA was not observed, and neutrophil and lymphocyte infiltration had improved. His renal function and urinary findings showed further improvement (eGFR: 50.8 ml/min.1.78 m^2^, proteinuria 1.25 g/gCr, CRP: 0.95 mg/dL) on hospital day 48. Then, we performed pulmonary and aortic valve replacement and ventricular septal closure on hospital day 53. Histopathological features of the replaced valves were consistent with infectious endocarditis. His postoperative course was uneventful, renal function further improved (eGFR: 64.3 ml/min.1.78 m^2^) on hospital day 63. The size of the multiple nodular shadows on chest computed tomography decreased, and he was discharged on hospital day 70. After that, his renal function maintained (eGFR: around 60 ml/min/1.73 m^2^ and proteinuria: <0.5 g/gCr) and PR-3 ANCA levels showed normal range on periodical examination in outpatient department.

## 3 Discussion

We report a case of rapidly progressive crescentic glomerulonephritis with histological features of GPA associated with infectious endocarditis. Our patient met the diagnostic criteria for GPA.^[4,5]^ Although several forms of renal disease have been reported to be associated with infectious endocarditis,^[6,7]^ to our knowledge, patients with crescentic glomerulonephritis accompanied with GPA are rare.^[1,8]^ Moreover, there are currently no guidelines for treatment of ANCA-positive glomerulonephritis associated with infectious endocarditis. Interestingly, Bele et al reported the success of combined treatment with antibiotics and immunosuppressants for systemic vasculitis, which may be caused by ANCA-associated infections.^[8]^ In contrast, others proposed treatment with antibiotics alone.^[9]^

In our patient, PR3-ANCA level was initially elevated, and it decreased with improvement of renal disease. PR3-ANCA is positive in 5% to 10% of patients with renal disease complicated with infectious endocarditis.^[6]^ PR3-ANCA may be produced as a consequence of an immune response against infection. Subsequently, it contributes to crescent formation, fibrinoid necrosis, and granuloma formation in the kidney.^[2,3,10–12]^ Renal diseases associated with infectious endocarditis have been shown to result in various pathological changes including crescent formation, fibrinoid necrosis, mesangial cell proliferation, endothelial cell thickening in glomeruli, and tubulointerstitial damage with infiltration of immune cells.^[2,3,11–13]^ A recent large cohort study that analyzed histological changes in kidney biopsies from 49 patients with infectious endocarditis-associated glomerulonephritis (regardless of ANCA positivity) revealed the most common histological change was necrotizing and crescentic glomerulonephritis (53%), followed by endocapillary proliferative glomerulonephritis (37%). C3 deposition was prominent in all cases, whereas IgG deposition was observed in <30% of cases.^[6]^ In the present case, we observed C3 deposition along glomerular capillary walls without deposition of immunoglobulins or electron-dense deposits in subendothelial and paramesangial areas. These findings suggest mechanisms associated with infectious endocarditis that are independent of PR3-ANCA contributed to development of renal disease in this patient. In contrast, other studies reported that immune complexes containing C3 can be deposited in glomerular capillary walls in patients with ANCA-associated glomerulonephritis without evidence of underlying infections.^[14,15]^ Further studies and case analyses are necessary to fully elucidate the mechanisms underlying the development of glomerulonephritis associated with infectious endocarditis, including the pathological roles of ANCAs and C3.

In the present case, pathogenic bacteria were not identified by blood cultures or culture test of the replaced valves. Increased serum levels of antistreptolysin O suggest possible *Streptococcus* species infection. However, we did not observe NAPlr staining or plasmin activity in kidney biopsies, which are histological markers for infection-related glomerulonephritis. We cannot exclude the involvement of infection, because NAPlr staining can be negative depending on time from disease onset to biopsy.^[16]^ C3 deposition in glomeruli observed by immunofluorescence and electron-dense deposits observed by electron microscopy may indicate infection-related glomerulonephritis. We were unable to diagnose the pathogenic condition related to the multiple nodular shadows on bilateral lung computed tomographic images because the patient declined to undergo lung biopsy. These may have been septic emboli or granulomas.

Regarding treatment for PR3-ANCA-positive renal disease complicated with infectious endocarditis, previous studies suggested antibiotic monotherapy for patients with low PR3-ANCA titers (<25 IU/mL) and combination therapy with antibiotics and immunosuppressive agents for patients with high PR3-ANCA titers (>50 IU/mL) when the condition does not improve with antibiotic monotherapy.^[17,18]^ In our patient, PR3-ANCA titer was low (11.0 IU/mL), and because of concern that immunosuppressive drugs would increase the risk of exacerbating infectious endocarditis, we initiated antibiotic monotherapy. The patient's PR3-ANCA titer normalized with improvement of renal disease. The results in our patient suggest antibiotic monotherapy can be effective for rapidly progressive crescentic glomerulonephritis with histological features of GPA associated with infectious endocarditis. However, histological evidence and more data regarding treatment outcomes are required for PR3-ANCA-positive renal disease associated with infectious endocarditis.

In conclusion, we report a case of PR3-ANCA positive rapidly progressive crescentic glomerulonephritis with histological features of GPA associated with infectious endocarditis. The monitoring of PR-3 ANCA level and repeated renal biopsy may be useful to evaluate the pathological condition of these cases.

## Author contributions

**Conceptualization:** Yoshiyuki Morishita.

**Data curation:** Momoko Hirata, Haruhisa Miyazawa, Junki, Morino, Shohei Kaneko, Saori Minato, Yanai Katsunori, Hiroki Ishii, Taisuke Kitano, Kiyonori Ito, Keiji Hirai, Takashi Oda, Akira Shimizu, Yoshihiko Ueda, Yoshiyuki Morishita.

**Writing – original draft:** Momoko Hirata, Haruhisa Miyazawa.

**Writing – review & editing:** Haruhisa Miyazawa, Yoshiyuki Morishita
